# Beneficial Effects of Combining Computed Tomography Enteroclysis/Enterography with Capsule Endoscopy for Screening Tumor Lesions in the Small Intestine

**DOI:** 10.1155/2015/952787

**Published:** 2015-02-22

**Authors:** Hiroaki Shibata, Shinichi Hashimoto, Kensaku Shimizu, Ryo Kawasato, Tomohiro Shirasawa, Takayuki Yokota, Hideko Onoda, Takeshi Okamoto, Jun Nishikawa, Naofumi Matsunaga, Isao Sakaida

**Affiliations:** ^1^Department of Gastroenterology and Hepatology, Yamaguchi University Graduate School of Medicine, 1-1-1 Minami-Kogushi, Ube, Yamaguchi 755-8505, Japan; ^2^Department of Radiology, Yamaguchi University Graduate School of Medicine, 1-1-1 Minami-Kogushi, Ube, Yamaguchi 755-8505, Japan

## Abstract

*Aim*. To compare the efficacy of using computed tomography enteroclysis/enterography (CTE), capsule endoscopy (CE), and CTE with CE for diagnosing tumor lesions in the small intestine. *Materials and Methods*. We included 98 patients who underwent CE during the observation period and were subjected to CTE at our hospital from April 2008 to May 2014. *Results*. CTE had a significantly higher sensitivity than CE (84.6% versus 46.2%, *P* = 0.039), but there were no significant differences in specificity, positive or negative predictive values, or diagnostic accuracy rates. The sensitivity of CTE/CE was 100%, again significantly higher than that of CE (*P* = 0.002). The difference in specificity between CTE/CE and CE was not significant, but there were significant differences in positive predictive values (100% for CTE/CE versus 66.7% for CE, *P* = 0.012), negative predictive values (100% versus 92.1%, *P* = 0.008), and diagnostic accuracy rate (100% versus 89.8%, *P* = 0.001). The diagnostic accuracy rate was also significantly higher in CTE/CE versus CTE (100% versus 95.9%, *P* = 0.043). *Conclusion*. Our findings suggested that a combination of CTE and CE was useful for screening tumor lesions in the small intestine. This trial is registered with number UMIN000016154.

## 1. Introduction

In terms of frequency, small bowel tumors have been considered rare in comparison with gastric and colon tumors; they have accounted for approximately 6% of primary gastrointestinal tumors and only approximately 1% of malignant gastrointestinal tumors [1–4]. Since 2001, there has been a widespread use of new techniques for examining small intestines, such as capsule endoscopy (CE) and balloon endoscopy (BE), and, as a result, small intestine tumors were found to account for a higher percentage than previously reported [5–8].

CE was first reported by Iddan et al. and has become the first choice for screening lesions in the small intestine [7, 9]. CE has mainly been reported to be useful in obscure gastrointestinal bleeding (OGIB) [10–12], but recent reports have also shown its usefulness in Crohn's disease [13–15]. Regarding neoplastic lesions, Ross et al. previously reported that the rate of diagnosing neoplastic tumor lesions in the small intestine by CE was 5/15 cases (33.3%) [16]. Zagorowicz et al. previously reported that the passage of CE over neoplastic lesions present in the proximal jejunum was fast; therefore, there was a risk of overlooking the diagnosis [17]. Thus, even when CE is used, diagnosing tumor lesions in the small intestine can be difficult in some cases, and the prognosis of small intestine malignancies is unfavorable [18–20]. It would be preferable to have tests that accurately diagnose such tumor lesions.

Thus far, using computed tomography (CT) to perform evaluations on the small intestine has been considered impossible; however, CT enteroclysis/enterography (CTE) has made it possible, because the small intestine can be expanded by using a negative contrast agent and images are obtained through contrast-enhanced CT [21, 22]. CT enteroclysis is a testing method that consists of injecting a negative contrast agent into the small intestine through a cannula [21], whereas, in CT enterography, the small intestine is expanded through the oral ingestion of a negative contrast agent [22]. While most reports on CTE have been about its diagnostic performance for the intestinal inflammation and intestinal complications of Crohn's disease [23], reports on the utility of CTE in the diagnosis of tumor lesions in the small intestine have also been found recently [24]. However, to date, there have been few studies that compare CE to CTE for evaluating neoplastic lesions [25–27]; in addition, there has been no study on the tests' sensitivity or specificity of neoplastic lesions or their rate of diagnostic accuracy. Therefore, we compared the diagnostic performance of CTE with that of CE in the assessment of tumor lesions in the small intestine, and we also examined the diagnostic performance of using a combination of the two tests.

## 2. Materials and Methods

### 2.1. Patients

The study was conducted retrospectively on 98 CE-tested patients who had been subjected to CTE at our hospital between April 2008 and May 2014. The male-female ratio was even (49/49 cases), and the mean age was 63.9 ± 16.5 years. The median duration of the time between CTE and CE was 2 days (range, 0–156 days). The indications for the tests were as follows: OGIB for 73 patients (74.5%); suspected neoplastic lesions for 12 (12.2%); Crohn's disease for 2 (2.0%); suspicion of Crohn's disease for 3 (3.1%); and other for 8 (8.2%) (Table 1). This study was approved by the Ethics Committee of Yamaguchi University Hospital.

### 2.2. Computed Tomography Enteroclysis/Enterography

CT enteroclysis was performed based on Liu et al.'s method [28]. A nasal endoscope was inserted into the duodenum. Next, a guide wire was inserted into the jejunum from the forceps port of the endoscope. After the nasal endoscope was removed, a 16-French balloon-tipped tube was inserted into the duodenojejunal flexure along the guide wire. After the balloon at the tip of the tube was inflated, 1200–1800 mL of a polyethylene glycol (PEG) solution, which had been warmed to about 37°C, was injected into the small intestine at 150 mL/min by using a pump. After the PEG solution was injected, the patient was immediately moved to the CT device, and a plain CT was performed followed by a contrast-enhanced CT. After the contrast agent for the contrast-enhanced CT was injected, three-phase imaging (40 sec, 70 sec, and 120 sec) was performed. Before the patient was moved to the CT device, butyl scopolamine (20 mg) was injected intramuscularly as an antispasmodic agent. When butyl scopolamine was contraindicated, glucagon (1 mg) was injected intramuscularly, and in patients for whom glucagon was also contraindicated, antispasmodic agents were not used. Tests using CT enterography were performed by referring to Huprich and Fletcher's method [29]. For 1 h, 1000–1800 mL of the PEG solution was ingested orally, and CT imaging was performed by using the same method as that used during CT enteroclysis.

The CT images were interpreted by a radiologist (Kensaku Shimizu) with >20 years of experience in interpreting CT images. After a conventional interpretation was performed, the CTE findings were confirmed once again by examining the CE findings. The purpose of the test was to examine whether the lesions could be depicted by CTE, not to examine whether the physician could diagnose the small intestinal tumor by the CTE findings.

### 2.3. Capsule Endoscopy

All patients were given an explanation about the complications associated with CE, such as an undescended or retained capsule, and were informed about the possible need for surgery in the case of a retained capsule, depending on the situation. Written informed consent was obtained for CE. The PillCam SB CE system (Given Imaging, Yokneam, Israel) was used, and the image interpretation was performed on a Rapid Reader (Version 6.5; Given Imaging). Each patient was instructed to fast for 12 h before the examination and was then asked to swallow the capsule. All patients were orally administered 40 mg of dimethicone syrup with a small amount of water before the examination to reduce air bubbles. After the examination began, the patients were allowed to drink water after 2 h and to eat light meals until it was confirmed that the capsule had arrived at the small intestine as indicated on the real-time viewer. Video recorders were collected until it was confirmed that the capsule had arrived at the large intestine on the real-time viewer, and the data were transferred to a workstation for analysis. CE findings were determined by a digestive endoscopy specialist (Shinichi Hashimoto) who had experience in interpreting CE data for >200 cases. After a conventional interpretation was performed, the CE findings were confirmed once more by referencing the CTE findings. The purpose of the test was the same as previously described for CTE.

### 2.4. Method for Evaluating Tumor Lesions in the Small Intestine

A definitive diagnosis of the tumor lesions in the small intestine was determined on the basis of findings from histopathological tests conducted on resected or biopsy specimens collected from BE or surgery. For patients who did not undergo BE or surgery, the final diagnosis was determined on the basis of clinical and imaging findings. The criteria for negative tumor lesions in the small intestine were as follows: (1) no tumor was detected by CE or CTE; (2) the presence of the tumor lesions was negative in BE and surgical findings; and (3) the symptoms believed to be due to neoplastic lesions were absent for ≥3 months after the tests.

### 2.5. Statistical Analysis

The chi-square test was used for the diagnostic rate of tumors. Statistical analysis was performed by using Ekuseru-Toukei 2012 (Social Survey Research Information Co., Ltd., Tokyo, Japan). Differences were considered statistically significant at a *P* value of <0.05.

## 3. Results

### 3.1. CTE and CE Findings

With CTE, the rate of positive findings was 52/98 cases (53.1%). Neoplastic lesions (i.e., polyps and submucosal tumors) were found in 13/98 cases (13.3%); suspicion of angioectasia, arteriovenous malformation, and other vascular lesions was found in 23/98 (23.5%); inflammatory findings (i.e., wall thickening, mucosal contrast enhancement, stenosis, fistulas, anal fistula, and perianal abscess) were found in 18/98 (18.4%); other lesions (i.e., abnormal small intestinal transit, suspicion of diverticulum, and postoperative transformation) were found in 3/98 (3.1%); and the findings were absent in 46/98 (46.9%).

With CE, the rate of positive findings was 75/98 cases (76.5%). Neoplastic lesions were found in 9/98 cases (9.2%); angioectasia was found in 25/98 (25.5%), mucosal injuries (i.e., reddening, erosions, ulcers, and ulcer scars) were found in 48/98 (49.0%); and the findings were absent in 23/98 (23.5%) (Table 2).

### 3.2. Small Bowel Tumor

Thirteen cases were finally diagnosed with neoplastic lesions, and in the remaining 85, the presence of tumor lesions of the small intestine was not confirmed on the basis of their clinical course and the tests that were conducted subsequently. In 11 of 13 cases, the tumors of the small intestine were detectable by CTE, and, in 6, they were detectable by CE. In all cases, small bowel tumors were detected by CTE and CE. However, a false-positive result was found in 2 of 13 cases with diagnosis of neoplastic lesions detected by CTE and 3 of 9 cases detected by CE. Gastrointestinal stromal tumors (GISTs) were found in 5 cases and were the largest in number. Various types of tumors such as neuroendocrine tumors (NETs), ectopic pancreas, and capillary hemangiomas were detected, but the primary small intestine cancers were not detected (Table 3).

**Figure 1:**
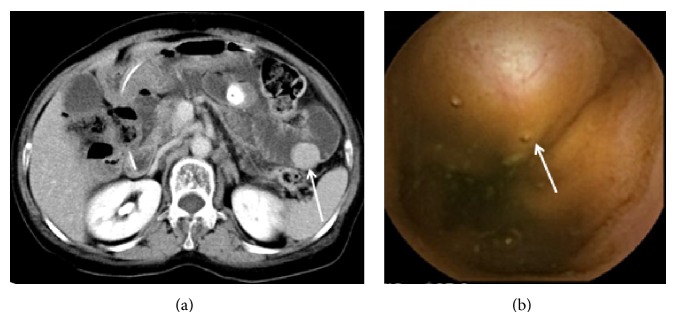
Findings from the computed tomography enteroclysis/enterography (CTE) and capsule endoscopy (CE). (a) Stained tumorous lesions in the small intestine detected by CTE and (b) submucosal tumor lesions confirmed by CE.

**Figure 2:**
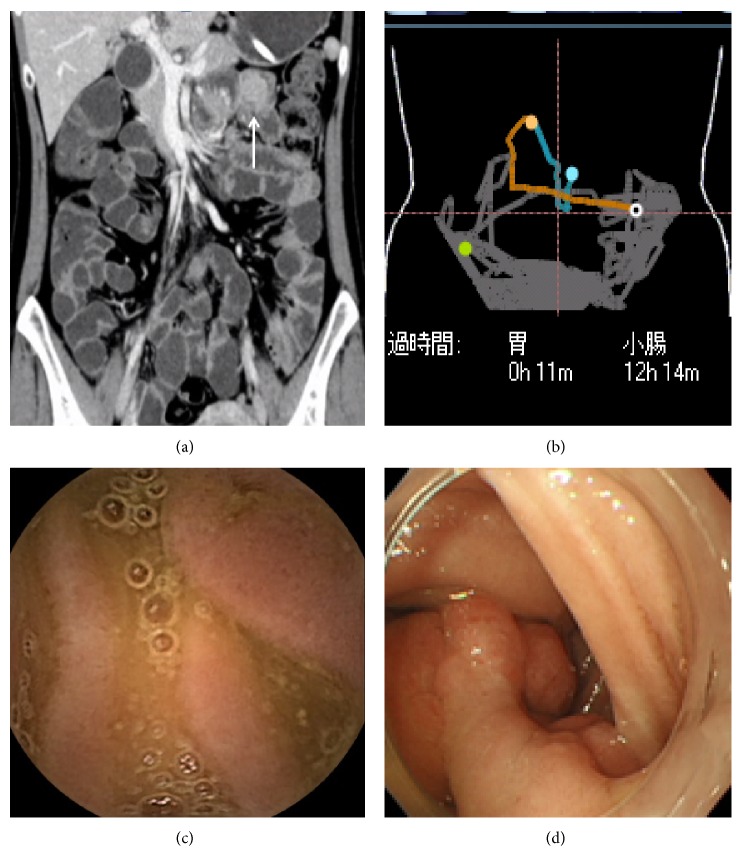
Findings from computed tomography enteroclysis/enterography (CTE), capsule endoscopy (CE), and balloon endoscopy (BE). (a) Strongly stained tumorous lesions in the small intestine detected by CTE; (b) the trajectory of CE in the vicinity of the area where CTE detected the lesions; (c) CE findings in the vicinity where lesions are detected by CTE. No lesion was detected by CE; (d) BE is performed after CE, and polypoid lesion was confirmed in the vicinity of the lesion detected by CTE.

Figure 1 shows a case in which the tumor lesions (GISTs) were detected by both CTE and CE and were treated surgically.  Figure 2 shows a case of Peutz-Jeghers syndrome; despite a tumor diameter >5 cm, the tumor could not be detected by CE. However, it was detected by CTE as a neoplastic lesion, and the tumor was then identified by BE; thus, polypectomy was performed.

The sensitivity to neoplastic lesions for CTE and CE was 84.6% and 46.2%, respectively, which was significantly different (*P* = 0.039). The specificity for CTE and CE was 97.6% and 96.5%, respectively (*P* = 0.650). The positive predictive values for CTE and CE were 84.6% and 66.7%, respectively (*P* = 0.323). The negative predictive values were 97.6% and 92.1%, respectively (*P* = 0.101), and the rates of diagnostic accuracy for CTE and CE were 95.9% and 89.8%, respectively (*P* = 0.096). Although the CTE was better than CE for diagnosing neoplastic lesions, the only significant difference was in sensitivity (Table 4).

In addition, the results of the tests using a combination of CTE and CE (CTE/CE) were compared to those using CE alone, and the findings showed that CTE/CE had a 100% sensitivity while CE had a 46.2% sensitivity (*P* = 0.002), which was significantly different. Specificity for CTE/CE and CE was 100% and 96.5%, respectively (*P* = 0.081). Although the specificity for CTE/CE was better than that for CE, the difference was not significant. The positive predictive values for CTE/CE and CE were 100% and 66.7%, respectively (*P* = 0.012); negative predictive values were 100% and 92.1%, respectively (*P* = 0.008); and the diagnostic accuracy rates for CTE/CE and CE were 100% and 89.8%, respectively (*P* = 0.001), all significant differences (Table 4). However, a comparison of the results from using a combination of CTE/CE to those from using CTE alone showed no significant difference in terms of sensitivity (*P* = 0.141), specificity (*P* = 0.151), positive predictive value (*P* = 0.141), or negative predictive value (*P* = 0.151). The diagnostic accuracy rates of CTE/CE and CTE were 100% and 95.9%, respectively, which was significantly different (*P* = 0.043) (Table 4).

## 4. Discussion

Compared to CE, our study findings showed that CTE had a higher performance for detecting tumor lesions in the small intestine. Additionally, the diagnosability of tumors increased even higher when CTE and CE were used together.

In our study, the sensitivity of CTE in the detection of small bowel tumor lesions was 11/13 cases (84.6%), and the sensitivity of CE was 6/13 (46.2%). These findings showed that CTE had a significantly higher sensitivity. A previous report by Hakim et al. also showed that the sensitivity of CTE in the detection of small bowel tumor lesions was 16/17 cases (94.1%) and that of CE was 6/17 (35.3%); therefore, CTE had a significantly higher sensitivity [26].

The particularly notable findings in this study were that, in as many as 7/13 cases (53.8%), small bowel tumor lesions could not be detected by CE. Similarly, Johanssen et al. also reported that the sensitivity of CE in the detection of small bowel NETs was 3/8 cases (37.5%) [25]. Huprich et al. also reported that when they conducted CE on OGIB patients, including 9 with small bowel tumor lesions, the diagnosis of small bowel tumors was overlooked in 3/9 cases (33.3%); therefore, they reported that, in some cases, small bowel tumor lesions can be overlooked by CE [30]. Thus, although CE has been considered as the first choice procedure for screening small bowel diseases [7, 9], its low sensitivity in the detection of small bowel tumor lesions may cause them to be overlooked. As a result of this, the best timing for therapeutic intervention may be missed.

Our study showed that, with CTE, we were able to detect 7 (100%) of the 7 cases in which small bowel lesions could not be detected with CE; meanwhile, with CE, we were able to detect 2 (100%) of the 2 cases in which neoplastic lesions could not be detected with CTE, suggesting that the combined use of CTE and CE would allow their abilities to complement each other and that the rate of overlooked small bowel tumor lesions using CE can be greatly reduced by combining CE with CTE.

No difference was found between CTE and CE in terms of specificity, positive predictive value, negative predictive value, or rate of diagnostic accuracy; however, the sensitivity with CTE was significantly higher than with CE.

A comparison of tests using a combination of CTE and CE to tests using CE alone revealed that a combination of CTE and CE showed greater significant differences in sensitivity, positive predictive value, negative predictive value, and rate of diagnostic accuracy. Therefore, in comparison with using CE alone for screenings, a combination of CE and CTE may be more highly reliable in diagnosing small bowel tumor lesions.

Our study also showed that small bowel tumor lesions might be missed by CE. It especially has been reported that for lesions in the jejunum in which a capsule moves quickly, submucosal tumors are easily missed [17, 31–34]. The issue that tumor detection depends on the transit rate may be resolved by the use of PillCam SB3, which automatically regulates the number of images according to the progress rate of capsules in the small bowel [35]. However, it is thought that submucosal tumors that grow outside the intestine may be difficult to detect by CE. By solving this issue, CTE may complement CE. However, there is the problem of radiation exposure in CTE. As less invasive tests to evaluate small bowel tumors, magnetic resonance enterography (MRE) and ultrasonography (USG) have been reported as useful to detect small bowel tumor-related lesions [36–38]. Nevertheless, various problems exist that MRE takes more time in comparison with CTE and diagnosis by USG depends on the skill of the diagnostician. Therefore, the prevalence of their use is lower than that of CTE and CE. Further examination using these modalities will be required in the future.

In addition, although BE can detect small bowel tumors, its performance for diagnosing is thought to be equivalent to CE. Furthermore, as a screening test, BE is a highly invasive procedure [34, 39].

The limitations of this study were the fact that it was retrospective and the number of cases was small. In addition, with regard to the rate of detection of neoplastic lesions by CE, an existing report used CE on 443 subjects, which detected small bowel tumors in 11 of the subjects (2.4%) [40]. In our facility, the number of small bowel tumors detected was as high as 6/98 (6.1%) and small intestine cancers, which accounted for 33–47% of primary small intestine tumors [4, 41], and malignant lymphomas, which accounted for 10–30% of malignant tumors of the small intestine [42], were not included in the details regarding the small bowel tumor lesions that were detected. Thus, the cases that were selected in our study may have been biased. Lastly, not all the cases underwent total enteroscopy using BE or surgical treatment; therefore, the findings pertaining to specificity, positive and negative predictive values, and rate of diagnostic accuracy maybe different from the actual situation.

However, to our knowledge, no report has mentioned the specificity, positive predictive value, negative predictive value, or rate of diagnostic accuracy with regard to the detecting small bowel tumor lesions. Therefore, our study is the first to examine this topic. Our findings indicate a need to conduct multicenter prospective studies in the future to further increase the diagnostic accuracy of small bowel tumor lesions.

## 5. Conclusions

Our study findings showed that the sensitivity for CTE in the detection of small bowel tumor lesions was significantly higher than that of CE. In comparison with the tests performed using CE alone, the combination of CTE and CE resulted in significant differences in terms of sensitivity, positive predictive value, negative predictive value, and rate of diagnostic accuracy. This suggests that CTE should be the first line of investigation in the screening for small bowel tumor and may be followed, if required, by CE.

## Figures and Tables

**Table 1:** Characteristics of the patients participating in the study.

Number of cases	98
Sex, male (%)	50
Age (years)	63.9 ± 16.5
Hb (g/dL) at the time when CTE was performed	9.4 ± 3.3
Time interval (median value) between CTE and CE	2 ± 26.3
Purpose of the tests	
OGIB	73 cases (745%)
Suspicion of neoplastic lesions	12 cases (12.2%)
Inflammatory bowel disease	5 cases (5.1%)
Others	8 cases (8.2%)

Hb: hemoglobin; CTE: computed tomography enteroclysis/enterography; CE: capsule endoscopy; OGIB: obscure gastrointestinal bleeding.

**Table 2:** Computed tomography enteroclysis/enterography (CTE) and capsule endoscopy (CE) findings.

CTE findings		CE findings	
Suspicion of angioectasia	17	Angioectasia	25
Tumor mass in contact with the small intestine	7	Ulcers	17
Enhanced contrast effect	6	Ulcer scars	2
Wall thickening	5	Sores	22
Stenosis	3	Reddening	5
Vasodilatation	3	Suspicion of stenosis	2
Suspicion of AVM	2	SMT	6
Suspicion of vasculitis	1	Stagnation	2
Suspicion of polyps	1	Polyps	3
Tumorous lesions	1	Blood	6
Densely stained punctiform lesion of the ileum	1	Diverticulum	1
Pedunculated mass in the proximal jejunum	1	Lymphangiectasia	1
Distension of the small intestine/abnormal small intestinal transit	1	No findings	23
Nonstained tumor mass inside the small intestine	1		
Suspicion of diverticulum	1		
Pouchitis	1		
Postoperative changes in the small intestine	1		
Tumor mass in contact with the small intestine/transverse colon	1		
Fistula	1		
Perianal abscess	1		
Anal fistula	1		
No findings	46		

AVM: arteriovenous malformations; SMT: submucosal tumor.

**Table 3:** Comparison of the computed tomography enteroclysis/enterography (CTE) and capsule endoscopy (CE) in terms of the final diagnoses.

Number	Sex/age	CTE	CE	Final diagnosis	Reference standard
1	F/55	No findings	Jejunal SMT	NET	BE, liver metastasis
2	M/74	Tumorous lesion in the ileum	Sores, ulcers	Ectopic pancreas	BE, surgery
3	F/58	Tumor mass in contact with the small intestine	Angioectasia	Small intestine GIST	Surgery
4	F/80	Densely stained punctiform lesion in the ileum	Sores, redness	Capillary hemangioma	BE
5	M/78	Tumor mass in contact with the small intestine	No findings	Small intestine GIST	BE, surgery
6	F/78	Tumor mass in contact with the small intestine	Sores, ulcers	Small intestine GIST	BE, surgery
7	F/25	Pedunculated mass in the jejunum	No findings	PJS	BE
8	F/55	Tumor mass in contact with the small intestine	No findings	Small intestine GIST	BE, surgery
9	M/72	Nonstained tumor mass in the small intestine	SMT	Lipoma	BE, surgery
10	M/72	Tumor mass in contact with the small intestine	SMT	NET	BE
11	M/59	No findings	Polyps	Cronkhite-Canada syndrome	CE
12	F/59	Tumor mass in contact with the small intestine	SMT	Small intestine GIST	BE, surgery
13	M/87	Tumor mass in contact with the small intestine	SMT	Metastatic small bowel tumor	CTE, CE, anamnestic

SMT: submucosal tumor; NET: neuroendocrine tumors; GIST: gastrointestinal stromal tumor; PJS: Peutz-Jeghers' syndrome; BE: balloon endoscopy.

**Table 4:** Comparison of computed tomography enteroclysis/enterography (CTE) and capsule endoscopy (CE) in terms of detecting the neoplastic lesions.

	CTE		CE		CTE/CE
Sensitivity		*P* = 0.039^*^		*P* = 0.002^*^	
	84.6%		46.2%		100%
			*P* = 0.141		

Specificity		*P* = 0.650		*P* = 0.081	
	97.6%		96.5%		100%
			*P* = 0.155		

Positive predictive value		*P* = 0.323		*P* = 0.012^*^	
	84.6%		66.7%		100%
			*P* = 0.141		

Negative predictive value		*P* = 0.101		*P* = 0.008^*^	
	97.6%		92.1%		100%
			*P* = 0.155		

Rate of diagnostic accuracy		*P* = 0.096		*P* = 0.001^*^	
	95.9%		89.8%		100%
			*P* = 0.043^*^		

^*^Significant differences.
